# Editorial: Word Morphology and Written Language Acquisition: Insights From Typical and Atypical Development in Different Orthographies

**DOI:** 10.3389/fpsyg.2019.00126

**Published:** 2019-02-06

**Authors:** Lynne G. Duncan, Daniela Traficante, Maximiliano A. Wilson

**Affiliations:** ^1^Psychology, School of Social Sciences, University of Dundee, Dundee, United Kingdom; ^2^Department of Psychology, Catholic University of Sacred Heart, Milano, Milan, Italy; ^3^Département de Réadaptation, Université Laval, Quebec, QC, Canada

**Keywords:** word morphology, developmental dyslexia, cross-linguistic perspective, literacy skills, morphological awareness training

By gathering together a body of work, across a range of orthographies, on the relationship between morphological processing and reading acquisition, an overview of the development of this relationship begins to emerge. The articles in this research topic offer insights into the relationship between morphological processing and semantics, that take account of the typicality of the acquisition process and the nature and depth of the orthography in question.

The literature on metalinguistic awareness illustrates the early salience and accessibility of meaning. In contrast, sensitivity to speech sounds, phoneme awareness (PA), usually requires specialist instruction to achieve the level necessary for acquiring the decoding skills essential for efficient reading in alphabetic orthographies (Duncan et al., 2013). Preliminary evidence suggests that with early morphological awareness (MA), statistical learning during the acquisition of spoken language may elaborate a system sufficient to underpin early reading subject to the linguistic characteristics of that language (Duncan et al., 2009).

Of the studies in this research topic that speak to this question, Colé et al. report on the first months of reading the French orthography by children from low socio-economic backgrounds. They show that MA affected PA both directly and indirectly (via listening comprehension) but had no direct impact on early word reading (see Diamanti et al. for further discussion of early shared variance between metalinguistic tasks). Schiff and Saiegh-Haddad consider the transfer between varieties in diglossic Arabic and the implications for instruction. Their cross-sectional study found that prior to 8th grade, MA was lower for morphemes which occur only in the formal standard Arabic of schooling than for morphemes which also occur in everyday spoken Arabic. Two studies of Greek examine the longitudinal impact of preschool MA developed from spoken language on early reading, after controlling for PA. Diamanti et al. found that Kindergarten MA predicted Grade 1 reading comprehension. However, it did not predict reading fluency, possibly due to the ease of decoding the transparent Greek orthography. As Greek is more opaque for spelling than reading, an additional predictive link with Grade 1 spelling was of particular interest. The larger scale study by Manolitsis et al. demonstrated that this longitudinal link between MA and reading comprehension (but not fluency) extends into Grade 2, even after further controls for autoregression, letter knowledge and RAN.

Several research topic articles explore the process of mapping the morphemes learnt during language acquisition onto corresponding orthographic patterns. Children's reading of complex words is known to benefit from the identification of embedded morphemes. Deacon and Francis showed that base frequency influenced reading in grades 3 and 5 beyond the number (family size) and the summed frequency (family frequency) of words sharing the same base. In other words, exposure to the meaning of the base word rather than to occurrences of its orthographic pattern may drive representation of embedded bases. Data from experiments using the masked priming procedure with a similar age group have also shown that the form-meaning relationship drives morphemic parsing in English (Beyersmann et al., 2012), French (Beyersmann et al., 2015), and Hebrew (Schiff et al., 2012). In this research topic, Lin et al.'s work offers interesting data from Korean Hangul, an alpha-syllabary language written in a nonlinear spatial layout (similar to Chinese). The study revealed an early priming effect (SOA = 36 ms) among 6th graders for truly morphologically-related pairs (e.g., bravely-brave), and provided new clues on the cross-linguistic validity of morphemic effects. Eye movements were used by Traficante et al. to examine primary school children's reading of derived words embedded in sentences. The results suggested that base-word recognition might affect word processing in a complex interplay with whole-word representation. Readers' skills, base-word grammatical category and syntactic context were shown to influence this interplay. Haddad et al. addressed the hypothesis that morphemic parsing supports phonological processes, particularly in languages with a deep orthography. Hebrew can be more or less transparent according to the presence or absence, respectively, of diacritics. In grades 2–5, morphological effects were only found with bimorphemic words written with diacritics. The authors concluded that morphological and phonological segmentation occur simultaneously in Hebrew, rather than being alternative pathways.

The identification of a base word within a complex word is thought to be particularly useful for struggling readers. For these children, the base word may act as a decoding unit of intermediate grain-size, larger than a grapheme and smaller than the whole word. Suárez-Coalla et al. reported that both Spanish primary school children with and without dyslexia take advantage of the presence of a high-frequency base when reading derived words and pseudowords in this transparent orthography. In Italian, another transparent language, Burani et al. focused on how the length of the first morpheme affected derived word processing. Although 6th graders with dyslexia displayed no length effects, their typically-developing peers showed faster reading of low-frequency derived words when bases were longer. Base frequency (but not whole-word frequency) influenced the reading latencies and accuracy of both groups. This confirms previous evidence that young readers use morphemic parsing in reading low-frequency derived words. A relationship between the ability to recognize morphemic constituents and reading proficiency was also found in Chinese. Kalindi and Chung demonstrated that MA uniquely predicted reading proficiency in adolescents with and without dyslexia, despite adolescents with dyslexia having lower MA.

From such evidence of morphological influences on literacy acquisition, it follows that reading might be improved through morphological training. Even though children are prone to focus their attention on the base word, Deacon and Francis propose that it may be necessary to teach them explicitly about the relationship between a word and its morphological family. Two further contributions also offer interesting suggestions about possible morphological interventions. Both works come from languages with a very rich morphology and a transparent orthography. Tsesmeli based her training for Greek 1st and 2nd graders on word families, offering explicit instruction on morphological decomposition. This training proved to be effective in improving the spelling of compound words. A computerized training in morphological analysis devised by Bar-Kochva and Hasselhorn enhanced spelling more than reading fluency or comprehension among 5 and 6th graders with literacy difficulties who speak German as a second language.

To conclude, this research topic provides cross-script and cross-linguistic evidence to further understanding of how sensitivity to the morphemic structure of the words develops from the pre-school to secondary school years, how this competence might help in literacy acquisition, and whether intervention methods focused on morphology can offer useful tools to improve literacy in struggling readers. Together, the evidence highlights a role for morphology that is particularly important for developing readers, whether they are struggling or typically developing. We are confident that this research topic on morphology will expand a promising but still emergent field of investigation and contribute to work that seeks to model the acquisition of complex word decoding and comprehension skills.

## Author Contributions

All authors listed have made a substantial, direct and intellectual contribution to the work, and approved it for publication.

### Conflict of Interest Statement

The authors declare that the research was conducted in the absence of any commercial or financial relationships that could be construed as a potential conflict of interest.
